# Levosimendan Administration in Limb Ischemia: Multicomponent Signaling Serving Kidney Protection

**DOI:** 10.1371/journal.pone.0163675

**Published:** 2016-09-29

**Authors:** Peter Onody, Peter Aranyi, Zsolt Turoczi, Rita Stangl, Andras Fulop, Emese Dudas, Gabor Lotz, Attila Szijarto

**Affiliations:** 1 1^st^ Department of Surgery, Semmelweis University, Budapest, Hungary; 2 2^nd^ Department of Pathology, Semmelweis University, Budapest, Hungary; 3 2^nd^ Department of Internal Medicine, Semmelweis University, Budapest, Hungary; Emory University Department of Medicine, UNITED STATES

## Abstract

**Aims and Objectives:**

Acute renal failure is a severe complication of lower extremity major arterial reconstructions, which could even be fatal. Levosimendan is a dual-acting positive inotropic and vasodilatory agent, which is suspected to have protective effects against cardiac ischemia. However, there is no data available on lower limb or remote organ ischemic injuries therefore the aim of the study was to investigate the effect of levosimendan on lower limb ischemia-reperfusion injury and the corollary renal dysfunction.

**Methods:**

Male Wistar rats underwent 180 min bilateral lower limb ischemia followed by 4 or 24 hours of reperfusion. Intravenous Levosimendan was administered continuously (0.2μg/bwkg/min) throughout the whole course of ischemia and the first 3h of reperfusion. Results were compared with sham-operated and ischemia-reperfusion groups. Hemodynamic monitoring was performed by invasive arterial blood pressure measurement. Kidney and lower limb muscle microcirculation was registered by a laser Doppler flowmeter. After 4h and 24h of reperfusion, serum, urine and histological samples were collected.

**Results:**

Systemic hemodynamic parameters and microcirculation of kidney and the lower limb significantly improved in the Levosimendan treated group. Muscle viability was significantly preserved 4 and 24 hours after reperfusion. At the same time, renal functional laboratory tests and kidney histology demonstrated significantly less expressive kidney injury in Levosimendan groups. TNF-α levels were significantly less elevated in the Levosimendan group 4 hours after reperfusion.

**Conclusion:**

The results claim a protective role for Levosimendan administration during major vascular surgeries to prevent renal complications.

## Introduction

Lower limb ischemic injuries (such as acute limb ischemia or even major vascular surgery on lower limb arteries) may represent serious clinical situations where profound cell destructions lead to substantial hemodynamic disturbances, systemic inflammatory changes and consequentially, severe multi-organ injuries [1]. Critical ischemic injury of the lower limbs might result in the loss of the limb or death, with mortality rates ranging from 15% to 20%.[2] Early detection of ischemia or deterioration of the injury is essential for survival. However, revascularization can result in further tissue damage due to reperfusion injury. A handful of surgical techniques and pharmacological approaches have been extensively investigated concerning the attenuation of IR injury.

Levosimendan is described as a dual-acting positive inotropic and vasodilatory agent developed for treatment of severe acute heart failure.[3] The agent exerts its positive inotropic effect through sensitizing the myocardium to Ca^2+^ by binding to and stabilizing the Ca^2+^ saturated troponin C.[4] The vasodilatory effect is related to the potential to open the ATP-sensitive K^+^ (K_ATP_) channels, localized in the sarcolemma of vascular smooth muscle cells in small resistance vessels[5] as well as in the venous side of the circulation[6], leading to reduced cardiac afterload and preload.[7] In the large conductance vessels, other (voltage-gated[8] and Ca^2+^-dependent[9]) subtypes of potassium channels have also been proposed to be opened by the drug though with equivocal clinical relevance.

Levosimendan has also been demonstrated to be an agonist of the mitochondrial type of K_ATP_ (mitoK_ATP_) channels.[10] Mitochondria play a central role in the pathogenesis of the I-R injury, and mitoK_ATP_ channels (located in the inner membrane of the organelle) have also been introduced as central mediator components in the ischemic protective methods. During ischemic preconditioning (IPC) and ischemic postconditioning (IPostC) several parallel signalling pathways converge at the level of mitochondria and lead to the opening of mitoK_ATP_ channels resulting in the stabilization of mitochondrial membrane thus limiting mitochondria mediated intracellular damage, therefore preventing cell death.[11–15]

Latest experimental data address some other effects of levosimendan, such as immunomodulatory, anti-inflammatory,[16] platelet aggregation inhibitory[17] and anti-apoptotic action.[18] The exact mechanisms of how the agent is able to achieve these positive effects are not yet fully understood, nevertheless these all may contribute to the cardioprotective and positive hemodynamic effects of the drug.

In spite of the great clinical importance of lower limb ischemic conditions, levosimendan previously has not been applied to limit injuries in such cases, therefore the aim of the present investigation was to apply levosimendan treatment in an experimental model of prolonged lower limb ischemia-reperfusion in which postoperative kidney dysfunction and systemic circulatory failure develop. Further goal was to assess the effects of the agent on local and systemic postischemic complications. Our previous investigations on the same model revealed that the technique of ischemic postconditioning can exert a protective effect on postoperative renal failure possibly through a mechanism involving mitoK_ATP_ opening,[19] which implies that levosimendan—acting on the same effector—might have similar effects.

## Materials and Methods

Male Wistar rats weighing 200–250 grams were used (Semmelweis University, Central Animal Facility, Budapest, Hungary). All investigations made on animals conformed to the guidelines of the US National Institute of Health on topic of use and care of experimental animals (Publication No. 85–23, revised 1996; MD, USA) also approved by the Committee on Animal Experimentation of Semmelweis University, Hungary (Permit No: 22.1/2409/3/2011).

Animals were kept under pathogen-free conditions, in 12-hour day-night cycles, at 22–24°C and had unlimited access to commercial pellets and water until 12 hours before operation, when only water was provided. Each experiment was performed at the same time of day to avoid any effects of circadian changes.

### Experimental design

**Fig 1:**
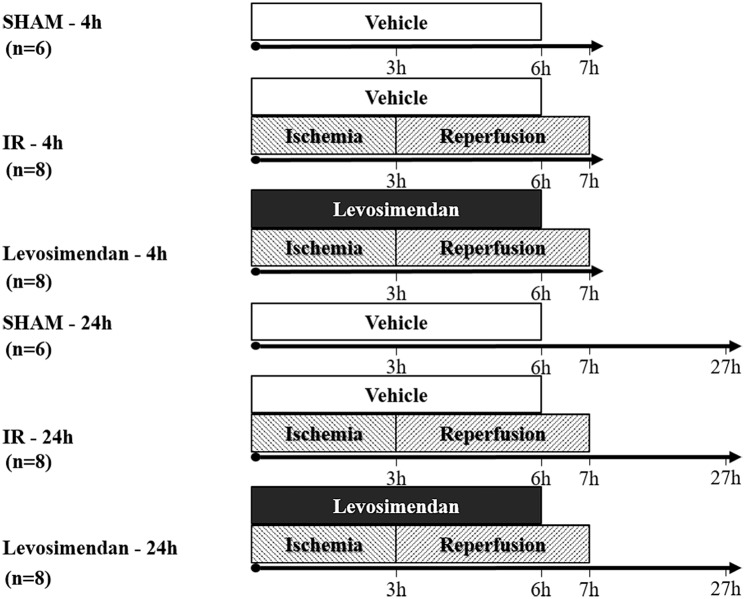
Study design and groups. Male Wistar rats underwent 180 min bilateral lower limb ischemia followed by 4 or 24 hours of reperfusion. Intravenous Levosimendan was administered continuously (0.2μg/bwkg/min) throughout the whole course of ischemia and the first 3h of reperfusion, while IR and Sham groups received only vehicle (Saline infusion).

Rats (n = 44) were distributed randomly into two main groups defined by the proposed length of reperfusion (4 and 24 hours), and were further divided into three subgroups according to the operation type (Sham-operation, IR and Levosimendan, 6-8-8 animals each group, respectively). (Fig 1)

Animals were anesthetized with intraperitoneal injection of ketamine (75 mg/bwkg) and xylasine (7.5 mg/bwkg). Deep anesthesia was maintained by continuous intravenous administration of anesthetics (ketamine and xylasine; 25 and 2.5 mg/bwkg/h respectively) and saline solution (3 ml/bwkg/h) via a 22-gauge polyethylene catheter (Harvard Apparatus, Holliston, MA) placed into the right jugular vein. Another polyethylene catheter was inserted into the right carotid artery connected to a blood pressure gauge to register mean arterial blood pressure (MAP) and heart rate (HR).

Through medianlaparotomy, the infrarenal section of the abdominal aorta was prepared. A laser Doppler flow probe (DP1T surface probe, Moor Instruments Ltd, London, UK) was placed on identical localizations on the ventral surface of the left kidney as well as on the surface of the left biceps femoris muscle through a 10 mm long incision on the skin and fascia as described previously.[19]

After a 30 minutes recovery period and baseline hemodynamic measurements, the infrarenal aorta was crossclamped with an atraumatic microvascular clip (Aesculap YASARGIL FT260T; B.Braun Melsungen AG, Melsungen, Germany), and the abdominal cavity was covered by a plastic wrap to prevent fluid loss by evaporation. 5 minutes prior to ischemia, 60 IU Na-heparin was administered intravenously. 3 hours of bilateral lower limb ischemia was established (IR and Levosimendan groups). At the end of ischemia, the microvascular clip was removed and reperfusion was allowed. (Animals in the 4 hours’ reperfusion group were kept in narcosis with further registration of hemodynamics and microcirculation. All other animals were allowed a survival time of 24 hours.) Animals were sacrificed at the indicated time points (4 or 24 hours after reperfusion) by exsanguination via right ventricular puncture, then muscle (right anterior tibial), right kidney and blood samples were collected.

Levosimendan (Simdax^®^ 2.5mg/ml, OrionPharma Ltd, Hungary), dissolved in 5g/100ml glucose solution, was administered as a continuous intravenous infusion (0.2 μg/bwkg/min, 0,48ml/bwkg/h with the dilution of 25 μg/ml, through the left jugular vein), initiated at the onset of infrarenal aortic clamping and throughout the whole course of ischemia and the first 3 hours of reperfusion (6 hours in total). In the first 10 minutes of administration, an increased (bolus) dose of 12 μg/bwkg levosimendan was given to mimic the clinically recommended administration protocol. IR and Sham-operated groups received only the vehicle (5g/100ml glucose solution) with the application of the same protocol.

Urine samples were collected throughout a 30 minutes’ baseline measurement period before ischemia to serve as a control. In animals subjected to 4 hours of reperfusion, another set of urine samples were collected from the start of the second hour on after reperfusion (i.e. for 3 hours) and their volume was measured. From the 24-hour-reperfusion animals, urine samples were taken through laparotomy by a needle puncture of the urinary vesicle.

Sham-operated animals underwent the same procedure described above except for the aortic occlusion.

### Systemic hemodynamics

Pressure signals were measured continuously (200 data per second) with a blood pressure gauge (Kent Scientific Corporation, Torrington, CT). A computerized data-acquisition system (DasyLab V9.00.02 software, National Instruments Corporation, Austin, TX) calculated and registered heart rate (HR), systolic (SBP) and diastolic (DBP) and mean arterial (MAP) blood pressure every 5 seconds.

Parameters underwent mathematical transformations as described previously by our group[19] using c++ (ISO/IEC14882 standard) code edited in Code::Blocks (10.05 rev6283m Code::Blocks Team, USA) and compiled by MinGW (2012., mingw.org). For eliminating the incidental measurement errors and failures due to external influences, some criteria were defined and enforced with the exclusion of data that failed these preset conditions. As criteria, upper and lower bounds for SBP, DBP and HR values were adjusted for each individual animal. Sudden changes in these parameters were also considered as results of external factors. Thus data were cancelled and replaced when exceeding a preset percentage of difference compared to the last accepted value. All invalidated and deleted data were replaced by the last known accepted values. When replacing multiple consecutive faulty values, the percentage interval of admissible parameters was increased exponentially with respect to the duration of the measurement error. The resulting sequences of data underwent further mathematical transformations (Gaussian smoothing) for the purpose of calculations and better comparability of the individual animals. For each parameter, a mean of nearby values weighted with Gaussian function was considered.

For detailed analysis of the blood pressure and heart rate data, the following calculations were performed:

1. Similarly to ‘shock index’, at each time point the quotient of HR and SBP was calculated and the mean was taken for both the period of ischemia and reperfusion. Dividing this mean of reperfusion by the mean of ischemia, a ratio was determined that is characteristic for the hemodynamic changes occurring after revascularization and the efficacy of the given animal’s compensative mechanisms.
2. To characterize and quantify the observed drop of blood pressure at revascularization, the lowest mean arterial pressure (MAP) was identified within the first 20 min of the reperfusion period. The time intervals were determined between this time point and the beginning of reperfusion, as well as the degree of this drop expressed as percentage of the last registered MAP of the ischemic period.
3. After the drop of blood pressure, a gradual rise was apparent in each animal reaching again a plateau state. With help of ‘IF function’ of Microsoft Excel (Microsoft Corporation, Redmond, WA, USA), the point in time was determined when mean arterial blood pressure reaches the beginning of the plateau phase.

### Microcirculation

Kidney and lower limb muscle microcirculation was measured by a laser Doppler flowmeter (LDF; Moor DRT4, 2 mW laser power at λ = 632.8 nm; DP1T surface probe, Moor Instruments Ltd, London, UK). Data were registered (at a sampling rate of 10/min) with the manufacturer’s software (MoorSoft for Windows v1.2, Moor Instruments Ltd, London, UK). To eliminate the variations among the baseline flow of the individual animals, all measured flux was normalized to and expressed as a percentage of mean of baseline flux. Two parameters were calculated: reperfusion area (RA, integral of the reperfusion segment of the graphs, proportional to the average blood-flow during reperfusion), and the plateau maximum (PM, mean of the last, plateau-shaped 10 min of the reperfusion-slope).[20]

### Histology

Muscle and kidney samples were fixed in 4% neutral-buffered formalin for a day, dehydrated and embedded in paraffin. Three μm thin sections were cut and stained with hematoxylin and eosin. Histological examinations were carried out with light microscopy (Olympus BX50 microscope equipped with Olympus DP70 camera; Olympus Corporation, Tokyo, Japan) by a trained pathologist in a blinded fashion. The pathological alterations were considered as follows: Muscle: (1) preservation of the striae of the skeletal muscle (2) preservation of the cell nuclei (3) intracellular vacuolisation (4) muscle fiber ripple, blurring (5) precipitation of the contractile proteins (6) homogeneity of cytoplasmic staining. Kidney:(1) glomerular condition (2) swelling of tubule cells, hydropic degeneration of kidney tubular epithelium and vacuolisation (3) precipitation in tubule lumens (4) presence of absorbed precipitate (5) detached epithelial cells (6) dilation of the vessels (stasis).

### Muscle viability

Part of the samples from the anterior tibial muscle were snap frozen in liquid nitrogen and stored at -80°C until further processing. 3 μm thick cross sections were cut in cryostat, then stained with NADH tetrazolium-reductase immonohistochemistry as described earlier.[21] Viability assessment was performed on the NADH-tetrazolium reductase stained muscles with the use of a morphometric method developed by our team.[22] The ratio of stained area and the total muscle fiber area was determined which correlates with the viability of the muscle fibers. The final result is expressed as a percentage of the values of untreated control muscles.

### Laboratory measurements

Serum (centrifuged) and urine samples were snap-frozen in liquid nitrogen immediately after harvest and stored at -80°C. Serum creatinine, carbamide, sodium concentration, urine volume, creatinine, and sodium concentration were measured. Analysis was performed by an automated clinical chemistry analyzer (Beckman Coulter AU480/2011, Beckman Coulter Inc, Brea, CA). Creatinine clearance ([Creatinine]_urinary_ x urine flow ÷ [Creatinine]_serum_), Serum [carbamide]/[creatinine] ratio, fractional sodium excretion (FENa) ([Na^+^]_urinary_ × [Creatinine]_serum_) ÷ ([Na^+^]_serum_ × [Creatinine]_urinary_) × 100) and renal failure index (RFI) ([Na^+^]_urinary_ × [Creatinine]_urinary_ ÷ [Creatinine]_serum_) were calculated.

Serum TNF α levels were assessed by sandwich ELISA kits (R&D Systems, Minneapolis, MN). Mouse TNF-α standard (200mL, in serially decreasing dilutions: 750–23.4 pg/mL), and 200 mL of TNF-α conjugate (polyclonal antibody to TNF-α conjugated with horseradish peroxidase) was added to each well in the ELISA microtiter plate, and incubated. A color reaction developed with a substrate containing equal volumes of stabilized hydrogen peroxide and tetramethyl benzidine (stopped after 20 min by addition of hydrochloric acid). Absorbance was measured at 450 nm. Each optical absorbance value was calculated as a percentage of the average optical absorbance.

### Statistical analysis

Data were analyzed using IBM SPSS Statistics Version 22 (IBM Corporation, Armonk, NY). All values are expressed as means ± S.D. For every group of animals the assumption of normality of each measured parameter’s distribution was tested using Kolmogorov-Smirnov statistic, then Levene’s tests for homogeneity of variances were performed. Accordingly, in cases when homogeneity of variances was assumed, one way ANOVA (analysis of variance) tests were calculated with Scheffe’s post hoc test for between groups comparisons. When heterogenity of variances was assumed, Brown-Forsythe test was performed with Games-Howell post hoc multiple comparisons. Any differences were considered statistically significant with p values less than 0.05.

## Results

### Systemic hemodynamics

No significant alterations could be found during the whole registration in the Sham operated group regarding both heart rate (HR) and mean arterial blood pressure (MAP). Blood pressure elevated significantly (p<0.001) after aortic occlusion and remained elevated throughout the period of ischemia in the IR group as compared to the Sham operated group. Levosimendan administration withheld this elevation until the 3^rd^ hour of ischemia. Reperfusion resulted in a drop in MAP in both groups (IR and Levosimendan) compared to the ischemic values.

**Fig 2:**
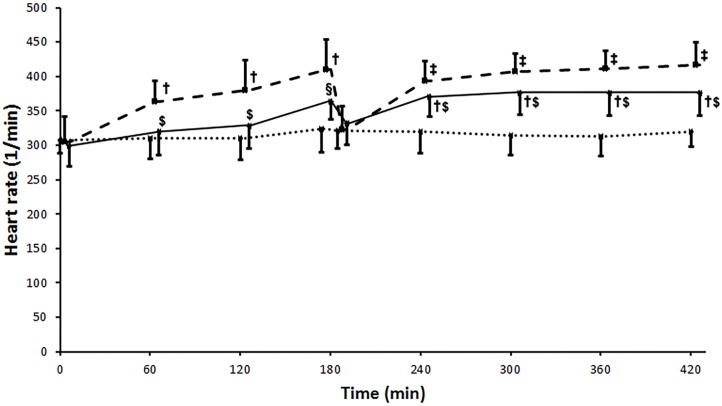
Heart rate. Heart rate increased significantly from the baseline during ischemia remained elevated during reperfusion in the IR (ischemia-reperfusion) group (broken line). Levosimendan administration resulted in significantly less elevated HR compared to the IR group during both ischemia and reperfusion (unbroken line). Sham operated group is marked with a dotted line. Values are expressed as means ± SD, †: p<0.01 vs. Sham, ‡: p<0.001 vs. Sham, $: p<0.05 vs. IR, §: p<0.01 vs. IR.

HR was significantly elevated during ischemia in the IR group, as measured after the 1^st^ (p = 0.004), 2^nd^ (p = 0.002) and 3^rd^ (p<0.001) hours versus Sham. Reperfusion resulted in another significant (p<0.001) increase in HR then remained elevated thereafter. Levosimendan administration resulted in significantly less elevated HR compared to the IR group both during ischemia and reperfusion (Fig 2) as measured after the 1^st^ (p = 0.016), 2^nd^ (p = 0.017), 3^rd^ (p = 0.023), 5^th^ (p = 0.049), 6^th^ (p = 0.038), and 7^th^ (p = 0.025) hours.

**Fig 3:**
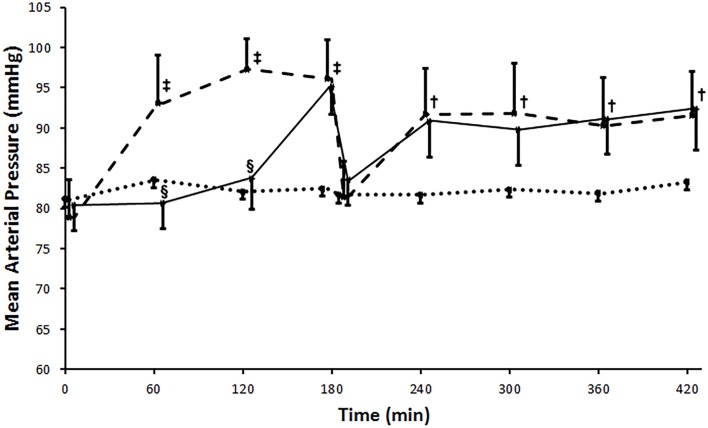
Mean arterial pressure. Blood pressure elevated significantly after aortic clamping and remained elevated throughout the ischemic period in the IR (ischemia-reperfusion) group (broken line). Levosimendan treatment withheld this elevation during ischemia (unbroken line). At the onset of reperfusion a significant drop occurred, but after that, mean arterial pressure increased and then remained elevated in both IR and Levosimendan groups without significant difference between the groups. Sham operated group is marked with a dotted line. Values are expressed as means ± SD, †: p<0.01 vs. Sham, ‡: p<0.001 vs. Sham, §: p<0.001 vs. IR.

1. Calculated ‘shock-index’ was higher for the period of reperfusion than for ischemia in the IR group (quotient >1). This was also the case in the Levosimendan group, with no statistically significant difference between the two groups (1.09±0.25 vs. 1.07±0.30, p>0.05).
2. The lowest systolic blood pressure within the first 20 min of reperfusion occurred at 6.00±2.86 min in the Levosimendan, and at 7.37±4.97 min after revascularization in the IR group, which did not differ statistically (p>0.05). This drop of blood pressure was greater in the IR group (16.61±3.61% of the last registered systolic blood pressure of the ischemic time) than in the Levosimendan group (8.56±8.22%), though because of the high standard deviation, the difference was found non-significant.
3. After the drop of the blood pressure, the pressure curves reached the plateau phase within a similar time interval in both the Levosimendan and the IR group (IR: 17.53±11.23 min vs. Levosimendan: 17.42±19.83 min after revascularization, p>0.05). (Fig 3)

### Microcirculation

**Fig 4:**
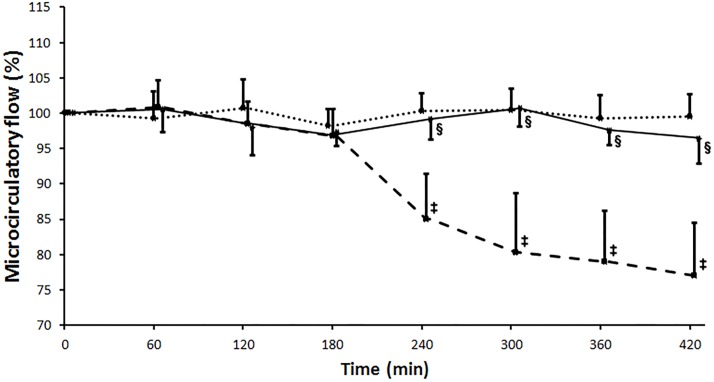
Kidney cortex microcirculation measured with laser Doppler flowmeter. Measured flux is expressed as a percentage of the baseline flux before the aortic clamping. Flux remained at the baseline level after clamping of the infrarenal aorta in all three animal groups. After revascularization of the limbs, a constant impairment of the kidney cortex microcirculatory flux was observed in the ischemia-reperfusion (IR) group (marked with a broken line), while flux was preserved in the Levosimendan (unbroken line) and the Sham (dotted line) groups. Values are expressed as means ± SD, ‡: p<0.001 vs. Sham, §: p<0.001 vs. IR.

Sham operation resulted in no alterations in either kidney or skeletal muscle flow throughout the experiment. Kidney microcirculation remained at the baseline level after clamping of the infrarenal aorta in both IR and Levosimendan groups. After revascularization, flux deteriorated gradually in the IR group while levosimendan administration was able to maintain renal cortical microcirculation at a significantly higher level (PM: IR: 78.02±12.63% vs. Levosimendan: 96.90±4.67%, p = 0.009; RA%: IR: 82.91±9.75% vs. Levosimendan: 98.84±1.85%, p = 0.004), resembling the values measured in the Sham operated group. (Fig 4)

**Fig 5:**
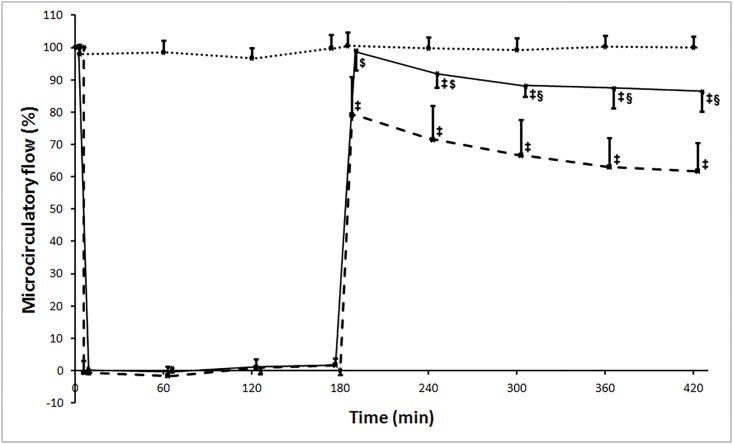
Muscle microcirculation measured with laser Doppler flowmeter. Measured flux is expressed as a percentage of the baseline flux before the aortic clamping. Flux dropped at the onset of aortic occlusion in both the ischemia-reperfusion (IR, marked with a broken line) and Levosimendan (unbroken line) groups. After release of the aortic occlusion, microcirculation increased to about 100% of baseline flux in the Levoismendan group, while in IR group flux reached only 80% of the baseline measurements. Sham operated group is marked with a dotted line. Values are expressed as means ± SD, ‡: p<0.001 vs. Sham, $: p<0.01 vs. IR, §: p<0.001 vs. IR.

Muscle microcirculation dropped at the onset of aortic occlusion in both the IR and Levosimendan groups. At the onset of reperfusion, microcirculation increased to about 100% of baseline flux in the Levoismendan group, in contrast to IR group where it reached only 80% of baseline values. Levosimendan treatment caused significant amelioration in every microcirculatory parameters. (PM: IR: 62.87±14.58% vs. Levosimendan: 89.25±6.70%, p = 0.004; RA%: IR: 66.01±14.92% vs. Levosimendan: 90.26±6.55%, p = 0.006). (Fig 5)

### Histology

Light microscopy and HE staining was not able to detect any definitive histopathological lesions in the samples taken from the tibial anterior muscle in any groups at any measured time points.

Kidney samples showed signs of acute tubular injury in the IR group with swollen, vacuolized tubular cells and precipitated hyaline-cylinders within the tubular lumina after 4 hours of reperfusion. 24 hours after aortic declamping slightly milder histological damage could be observed in the IR group compared to 4 hours of reperfusion. Levosimendan administration resulted in a lesser degree of histological injury at both examined reperfusion intervals compared to the corresponding IR group (qualitative analysis).

### Muscle viability

**Fig 6:**
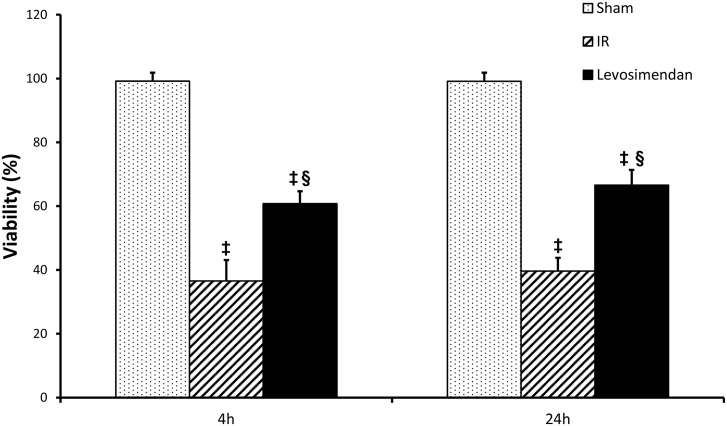
Muscle viability. 4 hours after revascularization a marked decrease in viability was detected in the IR (ischemia-reperfusion) group, without any sign of regeneration process 24 hours after reperfusion. Levosimendan administration resulted in significantly preserved viability compared to the IR group at both measured time points (4 and 24 hours). Values are expressed as means ± SD, ‡: p<0.001 vs. Sham, §: p<0.001 vs. IR.

4 hours after revascularization, a marked decrease in viability was detected in the IR group, as well as results showed no regeneration even after 24 hours of reperfusion. Levosimendan administration resulted in significantly preserved (p<0.001) viability compared to the IR group at both measured time points (4 and 24 hours), however the values of the Levosimendan group failed to reach the levels of the sham operated animals. (4 hours: Sham 99.203±2.625, IR: 36.601±6.507 vs Levosimendan: 60.746±3.932 p<0.001; 24 hours: Sham: 99.164±2.663, IR: 39.666±4.097 vs Levosimendan: 66.593±4.732 p<0.001). (Fig 6)

### Laboratory measurements

At the end of the fourth hour of reperfusion, kidney injury induced renal hypofunction was evident in the IR group according to markedly elevated serum creatinine levels (p<0.001), as well as creatinine clearance rates (p<0.001), and reperfusion urine volumes also showed a significant (p = 0,0??) deterioration of renal function compared to the sham operated group. Meanwhile in the 4h survival levosimendan treated group, significantly (p = 0.006) less expressive kidney injury could be observed.

Serum carbamide/creatinine ratio and the fractional sodium excretion indicated a tubular type kidney failure in the IR group, whereas the values in the Levosimendan group were not characteristic for any type of kidney failure, according to regulardefinitions. Differences were significant (p<0.001). Renal failure index and fractional sodium excretion indicated persistent kidney damage at the 24th hour after reperfusion with significantly lower values in the Levosimendan group (p<0.001) (Table 1).

**Table 1:** Laboratory measurements and calculated parameters for kidney function.

	Sham 4h	Sham 24h	IR 4h	Levo 4h	p (IR vs Levo)	IR 24h	Levo 24h	p (IR vs Levo)
**Creatinine[μmol/l]**	30.912±4.449	29.675±8.781	97.850±25.023	54.025±5.506	0.001	59.037±10.668	49.287±4.854	0.04
**Carbamide[mmol/l]**	4.46±0.665	4.965±0.602	5.908±1.217	5.516±1.001	0.49	6.462±0.894	5.837± 0.396	0.102
**Carbamide/ Creatinine**	148.033±34.804	183.492±69.417	62.599±14.496	101.837±13.114	0.001	112.821±25.922	119.568±15.413	0.539
**FENa [%]**	0.582±0.103	0.588±0.320	2.011±0.540	1.198±0.228	0.001	1.774±0.299	1.188±0.189	0.001
**RFI [mmol/l]**	0.799±0.076	0.801±0.239	2.616±0.387	1.657±0.168	0.001	2.333±0.204	1.611±0.157	0.001
**Creatinine clearance**	1.961± 0.596	-	0.199± 0.062	0.587± 0.165	0.001	-	-	-
**Urine volume**	1.013± 0.300	-	0.438± 0.177	0.738± 0.192	0.006	-	-	-

At the end of the fourth hour of reperfusion kidney injury was evident in the IR group. In contrast to Levosimendan group, where all of the measured and calculated kidney function parameters showed significantly less expressed kidney injury. Renal failure index (RFI) and fractional sodium excretion (FENa) indicated persistent kidney damage at the 24th hour after reperfusion with significantly lower values in the Levosimendan group. Values are given in each subgroup as means ± SD. Abbreviations: FENa: fractional sodium excretion. RFI: renal failure index

**Fig 7:**
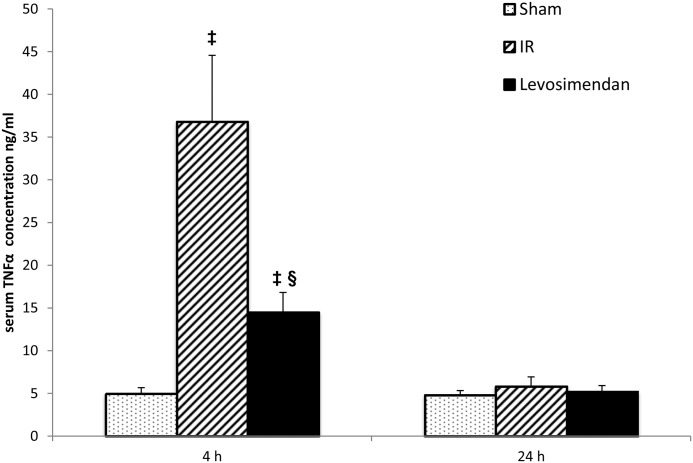
TNF-α concentration. TNF-α concentrations were significantly higher in the IR (ischemia-reperfusion) group compared to the Sham-operated group after 4 hours of reperfusion. TNF-α levels were significantly less elevated by Levosimendan treatment after 4 hours of reperfusion. 24 hours after reperfusion there were no significant differences among the three experimental groups. Values are expressed as means ± SD, ‡: p<0.001 vs. Sham, §: p<0.001 vs. IR.

TNF-α concentrations were significantly increased in the IR group compared to the Sham-operated group (p<0.001) after 4 hours of reperfusion. Levosimendan administration was able to significantly reduce serum TNF-α levels compared to the IR group at this measurement point (p<0.001). After 24 hours of reperfusion no differences (IR vs. SHAM:???; IR vs. Levo: p = 0.225) could be observed among the three experimental groups in serum TNF-α concentrations (Fig 7).

## Discussion

Prolonged lower limb ischemic conditions like acute critical limb ischemia with surgical revascularization therapy as well as major vascular surgeries of the lower limb arteries with crossclamping of the abdominal aorta might result in profound ischemia-reperfusion injury of the skeletal muscle tissues. The serious injury of the skeletal muscle involves the risk for postoperative systemic complications such as multiple organ failure.[23] The kidney is one of the most exposed organ in this process, with serious clinical implications of postoperative kidney dysfunction or even failure.[1] Many surgical and pharmacological techniques have been introduced to minimize the degree of ischemia-reperfusion injury. Levosimendan may be a reasonable alternative of these approaches, considering the already known fair tolerability, safety and well explored side-effect profile.[24, 25]

Besides its documented calcium sensitizer, positive inotropic and vasodilator properties mainly used in severe low output heart failure,[3] levosimendan has also been demonstrated to have a protective effect against ischemia-reperfusion injury via prevention of mitochondrial Ca^2+^ overload,[26] and opening of K_ATP_ channels in the inner membrane of mitochondria.[10, 15]

The aim of our present study was to investigate the impact of levosimendan in a lower limb IR model—that was previously utilized by our team—on local (muscle injuries) and systemic (kidney failure) complications.[19]

In the present study levosimendan treatment was demonstrated to have a protective effect against local IR injuries as evidenced by the skeletal muscle viability and muscle microcirculation measurements. Hematoxylin and eosin staining under light microscopy failed to reveal any detectable morphological changes in this model. For assessment of subcellular lesions and alterations in mitochondrial integrity, the viability of muscle fibers was assessed with a technique developed by our group.[22] Lower limb IR led to a significant reduction in the viability of muscle cells, while levosimendan administration was able to preserve muscle viability compared to the IR group.

In preservation of mitochondrial integrity by levosimendan, a couple of mechanisms may be involved. Levosimendan is a potential activator of the ATP-sensitive potassium channels (mitoK_ATP_) of mitochondria[27, 28]. Opening of mitoK_ATP_ activates distal signaling pathways, that involve improved defence against reactive oxygen species, protein-kinase Cε (PKCε),[12] mitochondrial connexin 43 (Cx43)[11], and reperfusion injury survival kinases (the RISK pathway),[27] which all converge for the prevention of opening of the mitochondrial permeability transition pore (mPTP). Opening of these channels is linked to mitochondrial dysfunction via dissipation of inner membrane potential, interruption of ATP synthesis, Ca^2+^ release, pyridine nucleotide depletion, matrix swelling and a consequential rupture of the outer membrane and release of proapoptotic proteins into the cytosol.[14]

The other important element of the protection of mitochondria is the regulation of cell’s calcium homeostasis. After a prolonged episode of ischemia, the Na^+^/Ca^2+^ exchanger (NCX) of the plasma membrane functions in a ‘reverse’ mode, resulting in Ca^2+^ influx to the cell. Overloading of calcium favors the pro-apoptotic Bcl2-associated X (BAX) protein incorporation into mitochondrial outer membrane that also leads to the opening of the mPTP.[29] It was shown by Li et al that administration of levosimendan inhibited the reverse mode of NCX activity, protecting the cadiomyocytes from Ca^2+^ overload.[26]

A less expressive IR injury in levosimendan treated animals was further confirmed by the finding of a considerably less impaired muscle microcirculation after levosimendan administration in contrast to the severely impaired muscle microcirculation in the IR group after revascularization. This finding can be explained at least in part by the vasodilating effect of levosimendan besides the positive impact on the limb tissues (muscle and a probable endothelial cell) IR injury. The protective effect of levosimendan on skeletal muscle viability and microcirculation shares similarities with our previous findings of the protective effects of ischemic postconditioning in the same model of limb IR injury.[19] Given the fact that levosimendan is a mitoK_ATP_ agonist and that the signaling pathways of ischemic postconditioning also involves the opening of the channel[11] it postulates a possible role of the channel opening in the effect of levosimendan behind the present results.

A protective effect against lower limb injuries would gain clinical relevance mainly by the impact it might have on the systemic complications of such injuries as for example the evolving kidney failure. Accordingly, renal dysfunction was characterized in the present model. We found that levosimendan administration resulted in milder histopathological alterations on kidney samples, less serious renal dysfunction (less elevated serum creatinine, minor oliguria and less impaired calculated GFR) at the early post-revascularization period, compared to the group of animals that received no levosimendan infusion. This nephroprotective effect was preserved even 24 hours after reperfusion. Calculated clinical functional laboratory parameters (fractional sodium excretion and serum carbamide/creatinine ratio) suggested a renal (tubular injury) type impairment of renal function in the IR group. The significant difference between the levosimendan treated and non-treated animals propose that levosimendan treatment results in less expressed acute tubular injury. This effect might be due to the ameliorated skeletal muscle IR injury, which resulted in less nephrotoxicity.

Our previous experimental and human investigations demonstrated a release of myoglobin from the injured myocytes after vascular exclusion of the lower limbs.[19, 30] On one hand, myoglobin interactions with Tamm-Horsfall proteins lead to tubular obstruction, on the other hand degradation and metabolism of myoglobin in tubular cells results in a heme-iron-mediated lipid peroxidation process.[31, 32]

Although there seems to be a direct relationship between the lower limb rhabdomyolysis and the acute kidney injury in our experiment, suggesting a direct toxic mechanism and an acute tubular necrosis type of renal failure, we proposed a prerenal injury beside the acute tubular necrosis. At revascularization of large skeletal muscles (previously exposed to hypoxia and with injured microcirculation and vasorelaxation of the larger blood vessels) serious hemodynamic changes evolve, even leading to shock, and a neuroendocrine response for circulatory redistribution, both effecting kidney circulation.[1] On one side, levosimendan has been described to improve perioperative cardiac performance[33] and may have beneficial effect in cardiogenic[34] and even in septic shock[35] presumably by its positive inotropic action. On the other side, by activation of vascular smooth muscle sarcolemmal K_ATP_ channels, this agent is known to evoke vasodilatation, a decrease in vascular resistance and mean arterial pressure. With detailed analysis of the systemic hemodynamic changes occurring in the present experimental model we could not find any significant effect when levosimendan is applied during a prolonged ischemia and revascularization of major lower limb arteries.

In contrast to the systemic hemodynamic parameters, infusion of levosimendan during limb ischemia and revascularization had a marked effect regarding renal microcirculation. The levosimendan treated animals presented a significantly less diminished cortical microcirculation during reperfusion as opposed by the considerable reduction of blood flow in the IR group. This circulatory impairment and the reduction of renal blood flow following skeletal muscle ischemia-reperfusion may arise from the systemic hemodynamic disturbances, the compensation of renal vasoconstriction, but also from the toxic oxidoreductive effects of myoglobin directly affecting renal microcirculation.[36] Levosimendan may increase renal circulation via preglomerular vasodilatation arising from its (sarcolemmal) K_ATP_ channel opening properties on vascular smooth muscle cells.[35, 37] All these contribute to the positive inotropy exerted on the right ventricle of the heart and a consequential reduction of the central venous and thus renal venous pressures.[38] Therefore, preglomerular vasodilation with decreased “right-sided” pressures lead to an improvement in renal blood flow and thus an increased GFR. Besides vasodilatation, levosimendan also attenuates the angiotensin II mediated mesangial cell contraction and thus increases the filtrating surface of glomeruli.[39]

Finally our results confirm the observations regarding the anti-inflammatory effect of levosimendan. TNF-α levels were significantly lower after 4 hours of reperfusion in the levosimendan group compared to the IR group. 24 hours after reperfusion no differences could be observed among the three experimental groups in serum TNF-α concentrations displaying the temporal characteristics of the cytokin elevation. Acute inflammatory changes and TNF-α may also contribute to acute renal failure,[40, 41] and levosimendan has been previously shown to exert anti-inflammatory effects,[42] the exact mechanisms of which are still not fully understood.

Our study presents some new results offering new perspectives for the use of levosimendan. We demonstrated that levosimendan treatment could protect muscle cells from ischemic injury after 3 hours of ischemia. In addition to these local effects, levosimendan reduced the degree of acute tubular kidney injury. An explanation of the positive effects may be the protection of mitochondria via opening the K_ATP_ channels and the amelioration of the skeletal muscle ischemia-reperfusion injuries, which may lead to the prevention of the myoglobinuric acute renal failure. The nephroprotective effect of the agent is further supported by the positive inotropic and hemodynamic effects of levosimendan on the level of microcirculation and the possible anti-inflammatory effect of the drug, confirmed in this study. These positive effects combined might result in the preservation of renal function.

### Limitation of the study

The study is mostly descriptive in nature and does not provide information/evidence about cellular changes during the process, most of the explanations and conclusions drawn are speculative. With purpose of standardization, specific pathogen free, healthy male Wistar rats were used only, so it is not suitable for detection the differences between genders and the population is not comparable for patients with high risk cardiovascular diseases. Moreover the anatomical differences (especially the circulatory system) makes the translation difficult, thus further, mainly big-animal studies are required to eliminate these differences.

## Supporting Information

S1 File Minimal Data Sets. Minimal data sets of the experimental material supporting ‘Levosimendan administration in limb ischemia: multicomponent signaling serving kidney protection’ article, including heart rate, mean arterial pressure, kidney cortex as well as muscle microcirculatory, muscle viability and labouratory measurements data, as broadly discussed individually at respective sections of the article.
(XLSX)
